# Supplementary material for: Comprehensive Insights into the Remarkable Function and Regulatory Mechanism of FluG during Asexual Development in Beauveria bassiana
Source: Int J Mol Sci. 2024 Jun 6;25(11):6261. doi: 10.3390/ijms25116261 (PMC11173134; doi:10.3390/ijms25116261)
Supplement: Supplementary file 1 [file ijms-25-06261-s001.zip › ijms-3011500-supplementary.pdf]

Comprehensive insights into the remarkable function and regulatory mechanism of FluG during asexual development in *Beauveria bassiana*

Fang Li[1], Haiying Zhong[1], Jianming Chen[1]*, Juefeng Zhang[1]*

[1] Institute of Plant Protection and Microbiology, Zhejiang Academy of Agricultural Sciences, Hangzhou, 310021, China

*** Correspondence:**

Jianming Chen, chenjm63@163.com;

Juefeng Zhang, zhangjf@zaas.ac.cn

Supplementary tables

Table S1 Paired primers used in manipulation of *fluG*, *brlA*, *abaA*, and *wetA* in *B. bassiana* and identification of their deletion/complement mutants.

Primers	Paired sequences (5′-3′) *	Purposes
FluGup-F/R	CCGGAATTCGTAAAGGCACTTGTCAATCCGC / CGCGGATCCGGTGGCAAGGGCAGTAAAATC	Cloning *fluG* 5′ (1599 bp) for deletion
FluGdn-F/R	CCGCTCGAGAGCATCCCAGCGATTCCAGT / GGAAGATCTTCTTTGGTGCGTGGTAGCCTC	Cloning *fluG* 3′ (1639 bp) for deletion
BrlAup-F/R	CCGGAATTCGGGGGAGAGAGACTATGGAATC / CGCGGATCCAAGAAGGTAAAGGCTGGTGA	Cloning *brlA* 5′(1544 bp) for deletion
BrlAdn-F/R	CTAGTCTAGACAATGTCAAGCCTGAACCTG / GGAAGATCTCCTCCTCGTAAAAGATAGCGT	Cloning *brlA* 3′ (1689 bp) for deletion
AbaAup-F/R	CGCGGATCCGAAGAGATGGAATAGATGAGGCA / CCCAAGCTTGCGTGTATGTGGTGTATGTGTGT	Cloning *abaA* 5′ (1695 bp) for deletion
AbaAdn-F/R	CCGCTCGAGACAGACAGGCACACAAGGATAC / GGAAGATCTTTGGAGAAGGAGTCAAACAGGT	Cloning *abaA* 3′ (1741 bp) for deletion
WetAup-F/R	CGCGGATCCAGACCTGGACAACGGAGAA / CCCAAGCTTGTTTGTTGGTGACCCTATGAG	Cloning *wetA* 5′ (1805 bp) for deletion
WetAdn-F/R	CTAGTCTAGAATGTATTCAACCACCAGCGG / GGAAGATCTAAGGCAATAAAAAGCCAGGG	Cloning *wetA* 3′ (1846 bp) for deletion
FluGfl-F/R	*GGGGACAAGTTTGTACAAAAAAGCAGGCT*TGTCTGTCCTACCTAAACGAGTC / *GGGGACCACTTTGTACAAGAAAGCTGGGT*CTCTTCCCACATTGCTAACG	Cloning full-length *fluG* (4590 bp) for complementation
BrlAfl-F/R	*GGGGACAAGTTTGTACAAAAAAGCAGGCT*AGTCTTGCCAGTCTCTTTTTG / *GGGGACCACTTTGTACAAGAAAGCTGGGT*TGTCATTTATTTGCGAGGTT	Cloning full-length *brlA* (4281 bp) for complementation
Abafl-F/R	*GGGGACAAGTTTGTACAAAAAAGCAGGCT*AGGACAGGGCAAAGAAGGTG / *GGGGACCACTTTGTACAAGAAAGCTGGGT*CTCTCCTCCTCCAACAACAGC	Cloning full-length *abaA* (5880 bp) for complementation
WetAfl-F/R	*GGGGACAAGTTTGTACAAAAAAGCAGGCT*ATTCCTCATTTCCTCATTGTC / *GGGGACCACTTTGTACAAGAAAGCTGGGT*GCTTGACCTGTCTGGATACC	Cloning full-length *wetA* (5234 bp) for complementation
idFluG-F/R	TCACAAAGAGGAAGGCGGTAT / CCTTGTTGACCTTCGGTATGC	PCR detecting of deleted /rescued *fluG*
idBrlA-F/R	TCAACTCTCTCTTGTTCACCAG/TAGATGCGTGGCACAACTTAC	PCR detecting of deleted/rescued *brlA*
idAbaA-F/R	CGATACAGACGCTTCATACCAGT/GCAAAGAGTAAAGATGTATTCGCT	PCR detecting of deleted/rescued *abaA*
idWetA-F/R	TGGTTGCCTGTTCTTCACTTTG/AGTGAGATGCCCTCCAGACG	PCR detecting of deleted/rescued *wetA*
RTidFluG-F/R	GGCAAGCGGTAGAGCAAC / GCGGAAACATTGTGGCTACC	RT-PCR detecting of deleted /rescued *fluG* (599 bp)
RTidBrlA-F/R	CTTTTCGTCTGCCTCTTCCG / CGTAGGAAACAGAGCAATAGGG	RT-PCR detecting of deleted/rescued *brlA* (469 bp)
RTidAbaA-F/R	CACCAGCCAACACAAGACTATG / TTTCCGCCCATGGAAAACTT	RT-PCR detecting of deleted/rescued *abaA* (482 bp)
RTidWetA-F/R	TGGCCTGACACCGACGTT / TGGGGTCAACATCATAGCCG	RT-PCR detecting of deleted/rescued *wetA* (280 bp)
18S-F/R	TGGTTTCTAGGACCGCCGTAA / CCTTGGCAAATGCTTTCGC	RT-PCR and qRT-PCR detecting
qFluG-F/R	CCATCAGGATACATCGTCTT / AGATTCATAGTCTCGCTCAA	qRT-PCR detecting of *fluG* transcript
qBrlA-F/R	GACCAGTTCAACAGACAAG / CAGTAATCTTCGTGCTTCTC	qRT-PCR detecting of *brlA* transcript
qAbaA-F/R	GCAAGTCTCCAGCCATAT / CTCCTCTTCGTCATAGTAGTC	qRT-PCR detecting of *abaA* transcript
qWetA-F/R	CGCAGACGAATTTGACTT/ GCTGGTGGTTGAATACAT	qRT-PCR detecting of *wetA* transcript

* The underlined regions are the introduced cleavage sites of restriction enzymes for the deletion of *fluG* (*Eco*RI/*Bam*HI and *Xho*I/*Bgl*II), *brlA* (*Eco*RI/*Bam*HI and *Xba*I/*Bgl*II), *abaA* (*Bam*HI/*Hin*dIII and *Xho*I/*Bgl*II), and *wetA* (*Bam*HI/*Hin*dIII and *Xba*I/*Bgl*II). The underlined and italicized regions are the recognition fragments for the gateway exchange in each complementary plasmid.

Table S2 Paired primers used in *B. bassiana* for confirming data validation of transcriptome by qRT-PCR.

Gene name/id	Paired sequences (5′-3′) *	Purposes
18S-F/R	TGGTTTCTAGGACCGCCGTAA / CCTTGGCAAATGCTTTCGC	qRT-PCR detecting
BBA_00001	TCTCAACTTCACAGTCTTC / CTGCCTGTCTGTATATCG	qRT-PCR detecting
BBA_00004	CCTCACCCAGAACTACTA / CCATCTCGTCAAGAATCTC	qRT-PCR detecting
BBA_00083	CAACTCCTACCGATTCTC / TTACCTATCGGCACATTG	qRT-PCR detecting
BBA_00193	CGACGGAGACAATAATACA / GCAGGACTGAAATACCAT	qRT-PCR detecting
BBA_00235	CAGGATTCCCTCCATCAC / AACTGGTCGTCGTAGTAG	qRT-PCR detecting
BBA_00319	GAGGTTGAGGAGATTGAG / GTGCTGTCGTAGATGTAG	qRT-PCR detecting
BBA_01324	AAGTCTGCTATCGCTTTC / CCGCATTCTCTATCAGTAG	qRT-PCR detecting
BBA_01327	CTTCGTCGTCTGATTCTT / AGTGGCTAGTGTAGATGA	qRT-PCR detecting
BBA_01509	GTGCTTTGCCATCATTAC / GCTCAACTCTACACTATCC	qRT-PCR detecting
BBA_02892	TGAAAGCCAACACCATTG / CCCTTGATAATCCCAGAAT	qRT-PCR detecting
BBA_03099	AATACCTTGGCATCCTTG / CTCTGGCTTAGTGTAGTC	qRT-PCR detecting
BBA_03642	GCCAATATCCTCATCGTT / GGTGATTCTCATCTTCCTT	qRT-PCR detecting

Figure S1

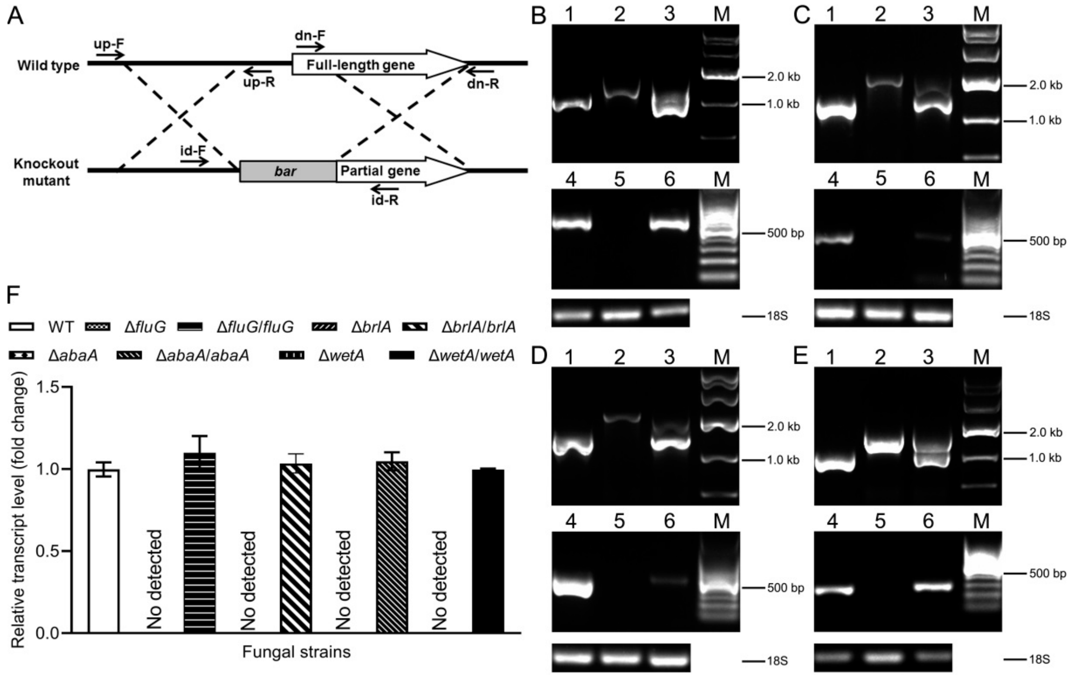

Figure S1. Constructing and identifying the single deletion of *fluG*, *brlA*, *abaA*, and *wetA* in *B. bassiana*. (A) Schematic diagram for the disruption of each gene. In knockout mutants, part of the coding sequence is replaced by the coding region of the *bar* gene with the promoter and terminator of *trpC*. (B, C) The mutants of *fluG* and *brlA* identified respectively via PCR (*lanes 1–3*) and RT-PCR (*lanes 4–6*). *Lanes 1* and *4*: wild type. *Lanes 2* and *5*: mutant. *Lanes 3* and *6*: complement. The bands of last row showed with internal reference 18S in different samples. (D, E) The mutants of *abaA* and *wetA* identified respectively via PCR (*lanes 1–3*) and Southern blotting (*lanes 4–6*). *Lanes 1* and *4*: wild type. *Lanes 2* and *5*: mutant. *Lanes 3* and *6*: complement. The bands of last row showed with internal reference 18S in different samples. (F) Relative transcript level of *fluG*, *brlA*, *abaA*, and *wetA* in the 4-day-old SDAY cultures of the deletion and complementation mutants versus the wild-type strain. Error bars: standard deviation of the mean from three cDNA samples of each strain detected in qRT-PCR experiments. See Table S1 for a list of paired primers used in PCR, RT-PCR and qRT-PCR.

Figure S2

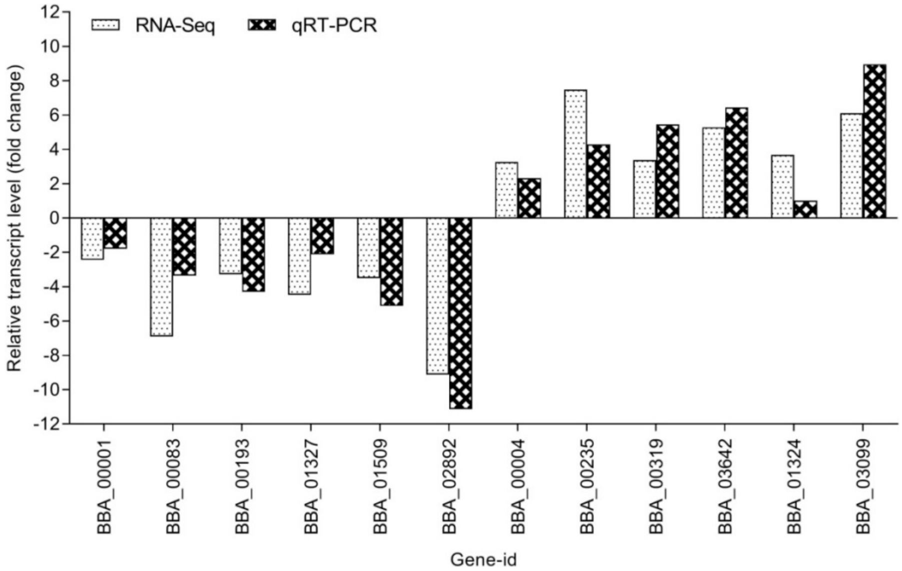

Figure S2. Comparison of gene expression patterns obtained by RNA-Seq and qRT-PCR. Changes in relative transcript levels of all 12 genes from the same sample were compared by transcriptome and qRT-PCR with paired primers in the Table S2.

Figure S3

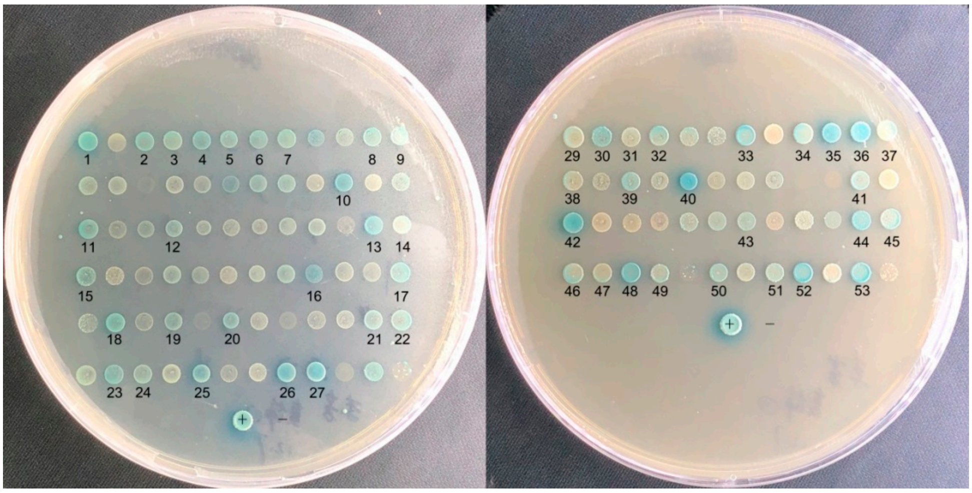

Figure S3. Positive clones on the re-screening plate. Pick the blue clones from the SD/-Leu/-Trp/-His/X-α-gal (TDO/X) screening plate and transfer them to SD/-Leu/-Trp/-His/-Ade/X-α-gal/AbA (QDO/X/A) plate for re-screening. 53 positive clones were successfully cloned and sequenced.

Figure S4

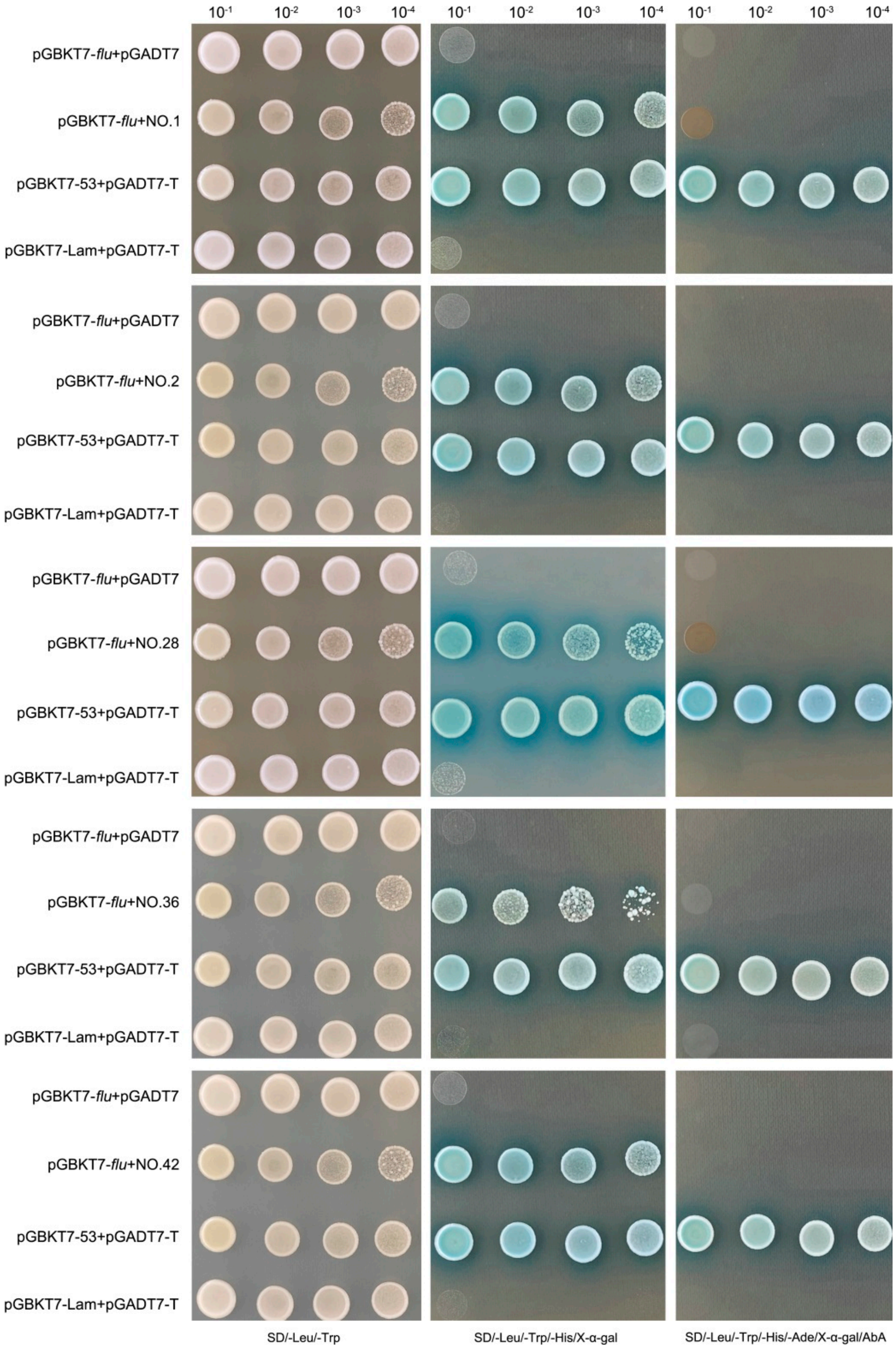

Figure S4. One - to - one yeast interaction validation. Five proteins were randomly selected to confirm the reliability of yeast interaction. The plasmids of pGBKT7-*fluG* and selected protein were used to confirm the interaction on different selection pressure plates.
